# Type 2 Endoleaks: The Diagnostic Performance of Non-Specialized Readers on Arterial and Venous Phase Multi-Slice CT Angiography

**DOI:** 10.1371/journal.pone.0149725

**Published:** 2016-03-01

**Authors:** Richard Nolz, Asenbaum Ulrika, Julia Furtner, Ramona Woitek, Sylvia Unterhumer, Andreas Wibmer, Alexander Prusa, Christian Loewe, Maria Schoder

**Affiliations:** 1 Medical University of Vienna, Department of Biomedical Imaging and Image-guided Therapy, Division of Cardiovascular and Interventional Radiology, Vienna, Austria; 2 Medical University of Vienna, Department of Biomedical Imaging and Image-guided Therapy, Division of General Radiology, Vienna, Austria; 3 Medical University of Vienna, Department of Biomedical Imaging and Image-guided Therapy, Division of Neuroradiology and Musculoskeletal Radiology, Vienna, Austria; 4 Medical University of Vienna, Department of Surgery, Division of Vascular Surgery, Vienna, Austria; University Francisco de Vitoria School of Medicine, SPAIN

## Abstract

**Purpose:**

To define the diagnostic precision of non-specialized readers in the detection of type 2 endoleaks (T2EL) in arterial versus venous phase acquisitions, and to evaluate an approach for radiation dose reduction.

**Methods:**

The pre-discharge and final follow-up multi-slice CT angiographies of 167 patients were retrospectively analyzed. Image data were separated into an arterial and a venous phase reading set. Two radiology residents assessed the reading sets for the presence of a T2EL, feeding vessels, and aneurysm sac size. Findings were compared with a standard of reference established by two experts in interventional radiology. The effective dose was calculated.

**Results:**

Overall, experts detected 131 T2ELs, and 331 feeding vessels in 334 examinations. Persistent T2ELs causing aneurysm sac growth > 5 mm were detected in 20 patients. Radiation in arterial and venous phases contributed to a mean of 58.6% and 39.0% of the total effective dose. Findings of reader 1 and 2 showed comparable sensitivities in arterial sets of 80.9 versus 85.5 (p = 0.09), and in venous sets of 73.3 versus 79.4 (p = 0.15), respectively. Reader 1 and 2 achieved a significant higher detection rate of feeding vessels with arterial compared to venous set (p = 0.04, p < 0.01). Both readers correctly identified T2ELs with growing aneurysm sac in all cases, independent of the acquisition phase.

**Conclusion:**

Arterial acquisitions enable non-specialized readers an accurate detection of T2ELs, and a significant better identification of feeding vessels. Based on our results, it seems reasonable to eliminate venous phase acquisitions.

## Introduction

Successful endovascular aneurysm repair of infra-renal aortic aneurysms (EVAR) is defined by the absence of an endoleak, which prevents aneurysm sac enlargement and rupture. Endoleaks are defined as persistent blood flow within the excluded aneurysm sac, and are classified based on the source of blood flow [1, 2]. Most endoleaks are type 2 endoleaks (T2EL), which are reported in up to 42% of patients with a rupture rate of 0.9% [3–5]. The management of T2ELs, i.e. watchful waiting vs. re-intervention [6], depends on whether the aneurysm diameter increases or not [7]. Multi-slice CT Angiography (MSCTA) is the imaging technique of choice for the diagnosis of endoleaks and aneurysm sac changes [7], and different imaging protocols have been suggested. While some authors have proposed a triple-phase protocol (i.e. unenhanced, arterial, and venous phase)[8], others showed that the arterial [9, 10] or venous phase [11] could be waived. However, bi-phasic MSCTA could miss low-flow T2ELs [12].

Currently, an estimated 50,000 patients per year undergo EVAR in the U.S. and Europe, respectively [13], with an annual increase of about 15% [14]. Given the mounting number of follow-up examinations, it becomes increasingly important that also non-specialized general radiologists are capable of adequately diagnosing T2ELs [15]. We therefore assessed the diagnostic performance of radiology residents with regard to T2EL detection, sac enlargement, and identification of feeding vessels in arterial and venous phase MSCTA. Based on these results, we evaluated an approach for radiation dose reduction.

## Materials and Methods

### Study Design

This was a retrospective study of patients being followed-up after elective EVAR of abdominal aortic aneurysm (AAA). The local ethics committee at the Medical University of Vienna, Austria (Local IRB NR: 1364/2015) approved the study protocol and waived written, informed consent. All patient data were anonymized and de-identified prior analysis.

### Study population

Our institutional database was screened for patients who met the following inclusion criteria: (1) elective endovascular AAA repair with a second- or third-generation bifurcated stent-graft; (2) availability of a standardized MSCTA post-procedural exam within one week, and after a time interval of at least six months. There were 215 patients who met the inclusion criteria. Patients with stent-graft-related endoleaks (n = 39), limb stenosis (n = 7), and a T2EL that caused continuous aneurysm sac enlargement necessitating secondary intervention within six months (n = 2) were excluded. This left 167 patients (15 female) with a mean age of 73.1 ± 8.4 years for final analysis.

### MSCTA Technique

MSCTA was performed in the caudo-cranial direction, using a 16-slice (Somatom Sensation 16, Siemens Healthcare, Erlangen, Germany; n = 138, 82.6%), or a Dual-source (Somatom Definition Flash; Siemens Healthcare, Forchheim, Germany; n = 29, 17.4%) scanner. The institutional standard protocol included an arterial phase, ranging from the celiac trunk to the groin, followed by a venous phase limited to the extent of the stent-graft.

A double-head power injector (Injektron CT2, Medtron AG, Saarbrücken, Germany) was used for intravenous injection, via an antecubital vein, of a non-ionic contrast medium (Iomeron 400, Bracco-Austria, Vienna, Austria), followed by a 40 ml saline chaser bolus. With the 16-slice CT scanner, arterial phase images were obtained biphasically during the injection of 30 ml and 85 ml Iomeron 400, at an injection rate of 6 ml/s and 4.5 ml/s, respectively. The scan was initiated six seconds after the attenuation of a region-of-interest positioned in the aorta at the level of the celiac trunk reached 110 HU (bolus-tracking technique). Dual-source MSCTA was performed after the injection of 110 ml Iomeron 400, at an injection rate of 6 ml/s, and was initiated 15 seconds after the threshold reached 150 HU. Venous phase with 16-slice scanner and Dual-source MSCTA was initiated 16 and 18 seconds after the arterial phase, respectively. Acquisition parameters are given in Table 1.

**Table 1:** Acquisition parameters.

	16-slice scanner	Dual-source scanner
**Tube voltage (kV)**	120	Ref 120 (Care kV)
**Tube current (refmAs, CD4D)**	120	120
**Collimation (mm)**	16 x 0.75	2 x 64 x 0.6
**Rotation time (sec)**	0.5	0.28
**Pitch**	≈ 1	≈ 1
**Soft kernel**	B30	B30

kV = kilovolt, refmAs = reference milliampereseconds, mm = millimeter, sec = seconds.

Post processing included thin maximum intensity projections and was performed from thin axial image slices on a Multimodality Workplace (MMWP, Siemens Healthcare, Erlangen, Germany) in the coronal and sagittal views, with a slice thickness of 3mm and a reconstruction increment of 2mm. All MSCTA images were transferred to the picture archiving and communication system (IMPAX, Agfa Healthcare, Mortsel, Belgium).

### Data evaluation and interpretation

Two residents with five years experience in general radiology retrospectively evaluated the pre-discharge and the final available follow-up MSCTA scans. MSCTA examinations were assessed in four reading sessions: (1) pre-discharge arterial phase; (2) pre-discharge venous phase; (3) final follow-up arterial phase; (4) final follow-up venous phase. The time interval between the reading sessions was two weeks. Patient order was randomly assigned in each reading session and for both readers using a computer generated list (www.randomizer.org). Readers were blinded to all clinical information. Images were assessed on a commercially available workstation (PACS system, version 5.2, AGFA-Healthcare, Mortsel, Belgium).

A T2EL was defined as a blush of contrast material in the aneurysm sac adjacent to an aortic branch (i.e., lumbar artery, inferior mesenteric artery, and accessory renal artery). The quantity of contrast-enhanced feeding vessels was noted. In addition, the maximum aneurysm diameter was measured perpendicular to the central lumen line, in accordance with the Society for Vascular Surgery reporting standards for EVAR [16] in arterial phase images.

Two physicians, with at least five years working experience dedicated to interventional radiology, who had direct access to all series of the pre-discharge and the final follow-up MSCTA scan, established the standard of reference in consensus. T2ELs identifiable on both MSCTA scans were classified as persistent; leaks detected only on one scan were classified as transient. Aneurysm sac diameter changes between the pre-discharge and the final follow-up MSCTA in each reading set were defined as a 4-mm difference in measurements, as proposed by Wever et al. [17]. Patients with a persistent T2EL and an aneurysm sac increase > 5 mm were classified as T2ELs that met the criteria for re-intervention [18, 19].

Reading sets were assessed by non-specialized readers for (1) detection of a T2EL, (2) number of feeding vessels, and (3) aneurysm sac diameter.

To assess the comprehensive detection of T2ELs on MSCTA images by non-specialized readers, the different reading sessions were pooled to an arterial and a venous set. All findings from the reading sets of non-specialized readers were compared to the defined standard of reference.

Findings from non-specialized readers resulting from a comparison of the pre-discharge and final follow-up MSCTA (e.g., T2EL perfusion status, sac diameters, and T2ELs meeting the criteria for re-intervention) were retrospectively analyzed.

### Dose calculation

To evaluate the radiation dose, the Dose Length Product (DLP) was calculated automatically by the scanner, based on the multiplication of the Computed Tomography Dose Index (CTDI) according to the scanned range. To determine the effective dose (E), we used the equation: E = E_DLP x DLP [20], where the E_DLP is a region-specific factor that is normalized to E. For the abdominal and the pelvic region, the E_DLP are 0.015mSv x mGy-1 x cm -1, and 0.019mSv x mGy-1 x cm -1, respectively. Since the scans in our study covered the abdomen and the pelvis, we used the mean value for both regions (E_DLP = 0.017mSv x mGy-1 x cm -1) to estimate the patient dose, as previously described by Macari et al. [9].

### Statistical analysis

All statistical analyses were performed using IBM SPSS Statistics Version 20.0. Metric data were presented as mean ± standard deviation or 95% confidence intervals when normally distributed, and as median and interquartile ranges (IQR) when skewed. Nominal data were presented as absolute frequencies and percentages.

Sensitivity, specificity, positive predictive value (PPV), negative predictive value (NPV), and accuracy were calculated for the detection of T2ELs, and T2ELs that met the criteria for re-intervention in each reading set, and for both readers. Comparisons between groups for sensitivities were performed using exact versions of the McNemar test.

Agreement of feeding vessel detection, and inter-reader agreement was described using Cohen’s weighted kappa. Comparisons of agreement between readers for dichotomous and polytomous variables were performed using exact versions of the McNemar test, the Friedmann test, or exact versions of the Wilcoxon matched pairs signed ranks test, as appropriate. Bland-Altmann Plots were used to compare aneurysm sac diameters between readers and the gold standard. Differences between aneurysm sac diameters in different groups were compared with an Analysis of variance. A p-value of ≤.05 was considered to indicate a significant result.

## Results

### Findings by experts

The mean follow-up was 32.0 ± 25.0 (IQR, 12.8–42.5) months. Overall, in 334 CT examinations, T2ELs were detected in 131 (39.2%) examinations. Overall, 331 feeding vessels were identified. During the observation period, 39 (23.4%) T2EL were transient and 46 (27.5%) persistent.

Overall, the aneurysm sac diameter was 60.1 ± 9.2 mm at the pre-discharge CT versus 56.3 ± 13.5 mm at the final follow-up CT, resulting in a diameter difference of -3.0mm (IQR, -10.0–1.0mm). Sac diameter was stable in 65 (38.9%), increased in 25 (15.0%), and decreased in 77 (46.1%) patients. Twenty patients (12%) with a persistent T2EL met the criteria for re-intervention.

### Non-specialized readers: arterial reading set

In arterial phase images, Reader 1 and 2 detected 120 and 109 T2ELs, respectively. Corresponding sensitivities for T2EL detection were 80.9 (95% CI: 72.9–87.1) for Reader 1 and 73.3 (95% CI: 64.7–80.5) for Reader 2, resulting in an inter-reader kappa of 0.795 (p = 0.071).

Reader 1 and 2 identified 300 and 269 feeding vessels (κ **=** 0.823, p < 0.001), respectively. When the detection of feeding vessels in arterial phase images was compared with the gold standard, both readers achieved a high agreement (reader 1: κ **=** 0.792; p< 0.001; reader 2: κ **=** 0.738; p< 0.001).

### Non-specialized readers: venous reading set

In venous phase images, Reader 1 and 2 found 127 and 117 T2ELs, respectively. Sensitivities for T2EL detection for Reader 1 and 2 were 85.5 (95% CI: 78.0–90.8), and 79.4 (95% CI: 71.3–85.8), with an inter-reader kappa of 0.794 (p = 0.110).

With regard to feeding vessels, Reader 1 and 2 found 256 and 219 branches (κ = 0.523; p < 0.001), respectively. When the detection rate of feeding vessels in venous phase images were compared with the gold standard, Reader 1 and 2 achieved a moderate agreement of κ = 0.483 (p< 0.001), and κ **=** 0.462 (p< 0.001), respectively.

### Non-specialized readers: Comparison of arterial and venous sets

A comparison of the arterial and venous phase images for the detection of T2ELs evaluated by Reader 1 and 2 is demonstrated in Table 2.

**Table 2:** Type 2 endoleak detection.

n = 334		Sensitivity	Specificity	PPV	NPV	Accuracy	p
**Reader 1**	**Arterial set**	80.9 (95% CI: 72.9–87.1)	93.1 (95% CI: 88.5–96.0)	88.3 (95% CI: 80.9–93.2)	88.3 (95% CI: 83.1–92.2)	88.3 (95% CI: 84.3–91.5)	0.092
	**Venous set**	85.5 (95% CI: 78.0–90.8)	92.6 (95% CI: 87.9–95.7)	88.2 (95% CI: 81.0–93.0)	90.8 (95% CI: 85.8–94.2)	89.8 (95% CI: 85.9–92.8)	
**Reader 2**	**Arterial set**	73.3 (95% CI: 64.7–80.5)	93.6 (95% CI: 89.1–96.4)	88.1 (95% CI: 80.1–93.2)	84.4 (95% CI: 78.9–88.8)	85.6 (95% CI: 81.3–89.1)	0.152
	**Venous set**	79.4 (95% CI: 71.3–85.8)	93.6 (95% CI: 89.1–96.4)	88.9 (95% CI: 81.4–93.7)	87.6 (95% CI: 82.2–91.5)	88.0 (95% CI: 83.9–91.2)	

PPV = positive predictive value, NPV = negative predictive value, p = sensitivity arterial set versus sensitivity venous set; statistical data in S1 Data.

Both readers achieved higher sensitivities for the detection of T2ELs on venous phase images, but without significant difference (p = 0.092, p = 0.152). However, using arterial phase images, Reader 1 reached a slightly higher sensitivity of 80.9 (95%CI: 72.9–87.1), compared to 79.4 (95%CI: 71.3–85.8) for Reader 2, using venous phase images (p = 0.839).

**Fig 1:**
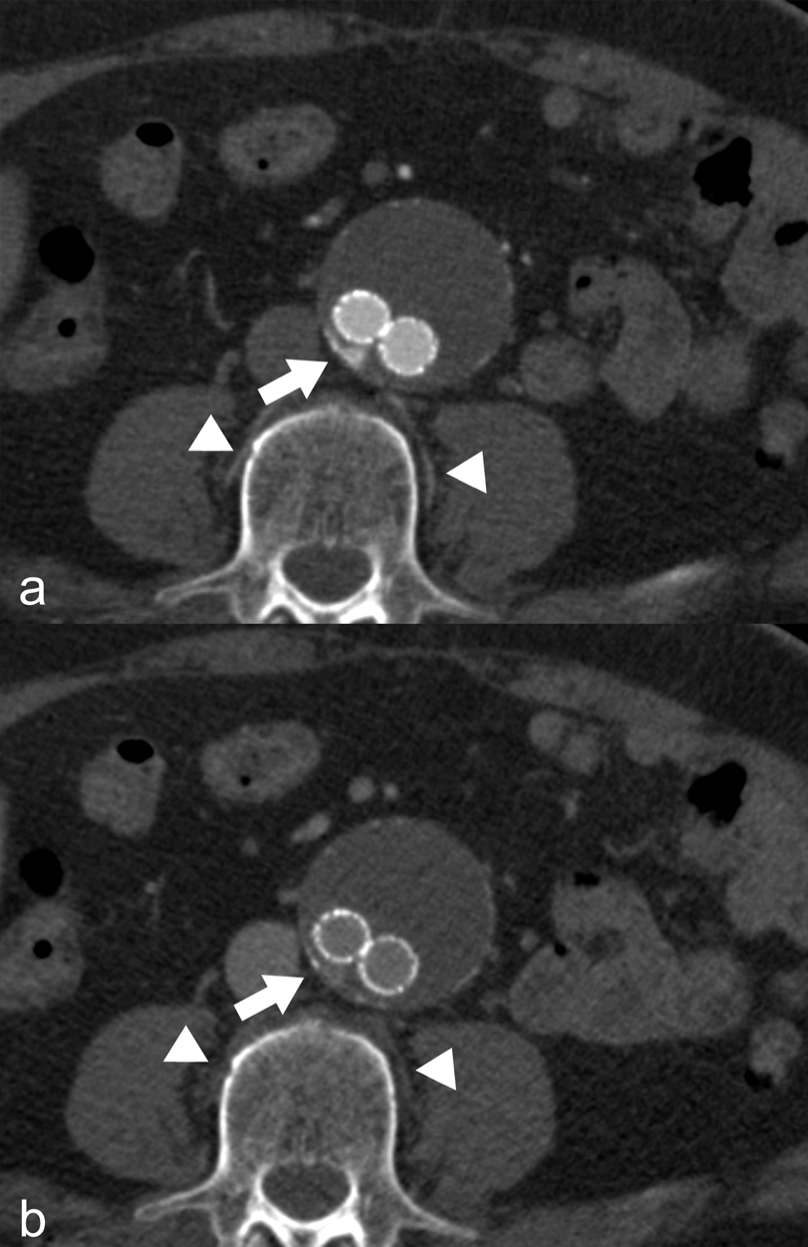
Feeding vessel detection in arterial and venous phase acquisition. (a) Arterial phase acquisition: Clearly perceptible blush of contrast agent in the aneurysm sac (arrow) indicative of the presence of a type 2 endoleak. Consecutive, feeding lumbar arteries of this segment show the same contrast as the aorta (arrowheads). (b) Venous phase acquisition: Similarly, clearly detectable type 2 endoleak (arrow). However, potentially feeding lumbar arteries show no contrast enhancement (arrowheads).

For both readers, detection rate of feeding vessels was significantly higher with arterial compared to venous phase images (Fig 1). Reader 1 and 2 achieved κ-values of 0.792 vs. 0.483 (p = 0.042), and 0.738 vs. 0.462 (p = 0.006), respectively.

### Non-specialized readers: Sac diameters

Aneurysm sac diameters for the gold standard and both readers, separated for the total study group and patients meeting criteria for re-intervention are described in Table 3.

Overall, no significant difference was found, when aneurysm sac diameters of Reader 1 and 2 were compared with the gold standard (p = 0.981).

**Table 3:** Sac diameters.

	Total (n = 167)			Type 2 endoleaks meeting criteria for re-intervention (n = 20)		
	Pre-discharge MSCTA	Last follow-up MSCTA	Sac diameter change	Pre-discharge MSCTA	Last follow-up MSCTA	Sac diameter change
**Gold standard**	60.1 ± 9.2mm	56.3 ± 13.5mm	- 3.0 (IQR: -10.0–1.0) mm	59.9 ± 7.0mm	71.9 ± 9.0mm	8.5 (IQR: - 7.3–13.8) mm
**Reader 1**	60.0 ± 9.5mm	56.1 ± 13.8mm	- 3.0 (IQR: -9.0–1.0) mm	60.4 ± 7.3mm	71.4 ± 9.8mm	7.5 (IQR: - 6.0–14.5) mm
**Reader 2**	60.1 ± 9.2 mm	56.3 ± 13.4mm	- 3.0 (IQR: -9.0–2.0) mm	60.1 ± 7.0mm	71.4 ± 9.0mm	8.0 (IQR: 6.0–13.0) mm

MSCTA = multi-slice computer tomography angiography, mm = millimeter, IQR = interquartile range; statistical data in S1 Data.

**Fig 2:**
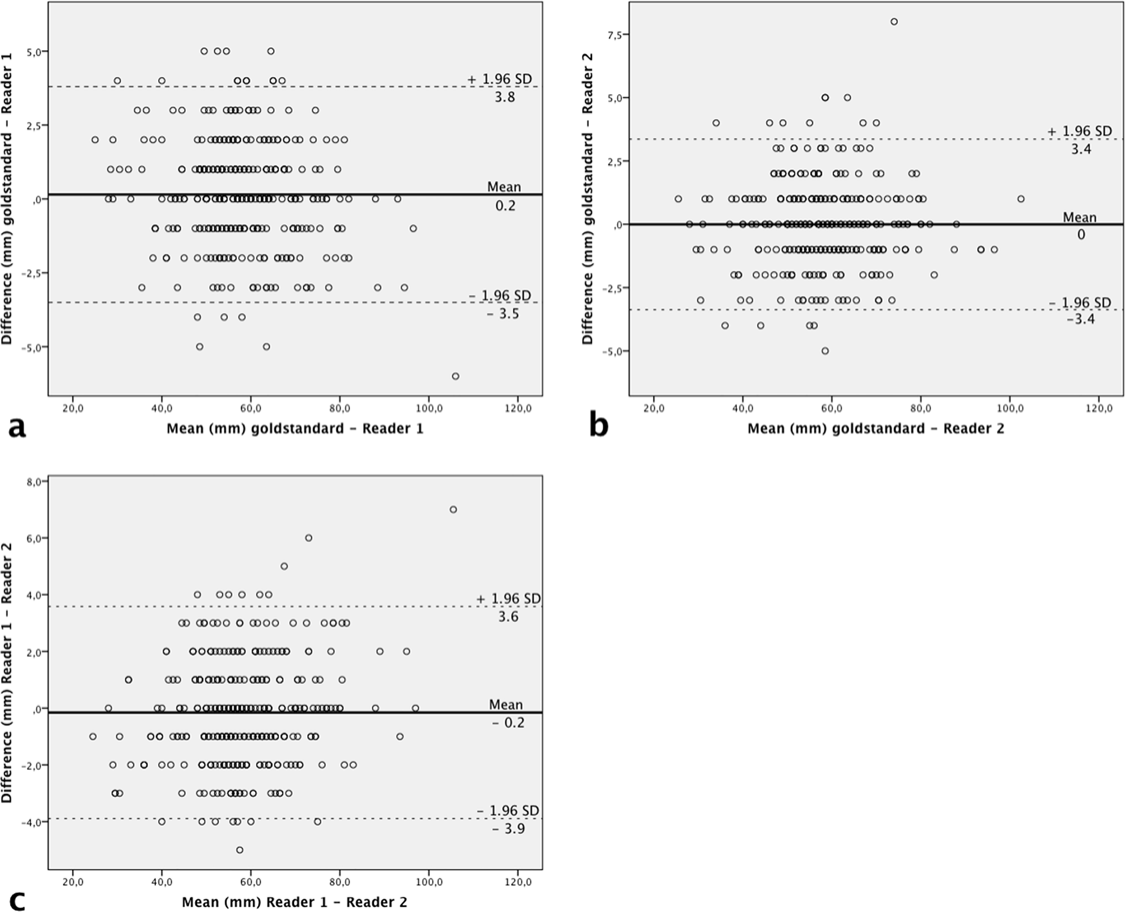
Scatterplots of diameter measurements of the aneurysm sac. Comparison of diameter measurements of the aneurysm sac (n = 334) between (a) gold standard and reader 1, (b) gold standard and reader 2, and (c) reader 1 and reader 2. The solid line denotes the mean of the differences (md) between compared groups. Dashed lines indicate the upper and lower limits of the agreement region (md ± [1.96 x SD]), where SD is the standard deviation of differences. mm = millimeter.

Mean differences of measurements of Reader 1 and 2 compared to the gold standard were 0.2 ± 1.9 mm, and 0 ± 1.7 mm, respectively. Inter-reader comparison revealed a mean difference of– 0.2 ± 1.9 mm (Fig 2A–2C).

### Diagnostic relevance of measurements

#### Arterial phase

An analysis of the readings of Readers 1 and 2 for the definition of perfusion status revealed persistent and transient T2ELs in 42 and 36, and 35 and 39 cases, respectively (k = 0.693, p < 0.001). Compared to the gold standard, readings of Reader 1 and 2 showed remarkable agreements of κ = 0.700 (p < 0.001), and κ = 0.620 (p < 0.001), respectively.

#### Venous phase

An analysis of readings of Readers 1 and 2 for the definition of perfusion status revealed persistent and transient T2ELs in 47 and 34, and 40 and 37 cases, respectively (κ = 0.735, p < 0.001). Compared to the gold standard, the readings of Reader 1 and 2 showed a remarkable agreements of κ = 0.750 (p < 0.001), and κ = 0.670 (p < 0.001), respectively.

For persistent T2ELs that met the criteria for re-intervention, both readers correctly identified all 20 cases in arterial as well as in venous phase images.

### Radiation dose

The average DLP for arterial and venous phase was 503.8 ± 257.4mGy*cm and 334.6 ± 183.9mGy*cm, respectively. The calculated effective dose was 8.6 ± 4.4mSv for arterial, and 5.7 ± 3.1mSv for venous phase. Therefore, arterial and venous phase contributed to a mean of 58.6%, and 39.0% of the total absorbed dose to the patient (Table 4).

**Table 4:** Dose calculations.

n = 289	Effective dose (mSv)	%	DLP (mGy*cm)
**Total**	14.6 ± 6.7 (95% CI: 13.8–15.4)	100	858.7 ± 395.3 (95% CI: 809.1–908.2)
**Bolus tracking**	0.4 ± 0.3 (95% CI: 0.3–0.4)	2.4	20.3 ± 16.5 (95% CI: 18.3–22.4)
**Arterial phase**	8.6 ± 4.4 (95% CI: 8.0–9.1)	58.6	503.8 ± 257.4 (95% CI: 471.5–536.0)
**Venous phase**	5.7 ± 3.1 (95% CI: 5.3–6.1)	39.0	334.6 ± 183.9 (95% CI: 311.5–357.6)

mSv = millisievert, DLP = dose length product, mGy*cm = milligray*centimeter, CI = confidence interval; statistical data in S1 Data.

## Discussion

In this study we found that for non-specialized readers, the detection of T2ELs and aneurysm sac enlargement were not different on arterial versus venous phase images. However, the identification of feeding vessels was significantly better on arterial phase images.

There is an ongoing controversy about the optimal imaging protocol for the detection of T2ELs after EVAR. Some authors have suggested acquiring both arterial and venous phase images, while others found it safe to waive one of them [8, 9, 11, 12, 21]. Rozenblit et al. [8] reported that compared to dual-phase acquisitions, only the combination of unenhanced, arterial and venous phase images was perfectly sensitive for the detection of T2EL and concluded that only the combination of all three phases enabled an accurate diagnosis. Other authors proposed reduced imaging protocols that would offer comparable diagnostic precision with reduced radiation exposure. Macari et al. [9] compared the arterial phase with a biphasic unenhanced and venous phase protocol and found T2EL detection rates of 22.7% versus 25.5%. Of all T2ELs, Bastos et al. [10] demonstrated a detection rate of 16.7% versus 26.7% when comparing arterial and venous phase images. Therefore, both authors recommended that the arterial phase could be eliminated, especially in stable or decreasing aneurysm sac sizes. Iezzi et al. [11], comparing arterial, unenhanced and arterial, and arterial and venous phase, found sensitivities of 80%, 90%, and 100%, respectively, but without significant differences. Based on these results, contrary to Bastos et al. [10] and Macari et al. [9], these authors suggested eliminating the venous phase. The T2EL-detection rate was higher with venous phase acquisitions, but a significant difference was not provided in any of these studies.

When we compared our findings to other results, venous phase images showed clearly lower sensitivities, whereas arterial phase images were comparable. The above-mentioned studies focused on the detection of endoleaks, but no attempts were made to correlate the presence of an endoleak with aneurysm sac changes, which may influence the decision for re-intervention. In the presence of a T2EL, several authors observed aneurysm sac enlargement in 24% to 55% of patients during midterm follow-up [4, 18, 22, 23]. In a previous study, we showed that the progression of the aneurysm sac was dependent on the perfusion status, with a significantly higher growth rate in persistent T2ELs [23]. However, a high spontaneous sealing rate of 62% was reported by Hong et al. [21], and included all T2ELs that were detected only in the venous phase. In addition, for transient T2ELs, stable or decreasing aneurysm sac diameters were found at a mean follow-up of 22–68 months [23, 24]. The T2EL detection rate and sensitivities were highest in a combined arterial and venous phase protocol. However, biphasic MSCTA protocols may fail to detect every T2EL [25, 26]. Recent dynamic CT studies demonstrated that endoleaks show a mean peak contrast enhancement after 9–27 seconds, which is in the detection range of a commonly used MSCTA arterial phase [25–27]. Regarding the detection of low-flow T2ELs, Iezzi et al. [12] demonstrated the superiority of late venous phase after 300 seconds, compared to commonly used venous phase after 60 seconds. The clinical relevance of T2ELs depends on aneurysm sac growth, which determines the need for re-intervention [18, 19].

Our results showed that non-specialized readers were able to detect every case of persistent T2ELs with increasing aneurysm sac diameters in arterial phase images. For the accurate planning of re-intervention on T2EL, the detection and localization of the feeding vessels is crucial. Furthermore, as shown by Dudeck et al. [15], the number of feeding aortic branch vessels influences the need for re-intervention.

In our study, non-specialized readers detected a significantly higher number of feeding vessels on arterial compared to venous phase images. This is confirmed by recent data from a dynamic MSCTA study, where peak enhancement of the aorta, as well as the aortic branches, was observed after 12 to 17 seconds [25].

Our data reinforce the strategy of eliminating the venous phase in order to reduce radiation dose, as earlier suggested by Iezzi et al. [11]. By eliminating the venous phase, a reduction of more than one-third of the total effective radiation dose can be achieved. Follow-up MSCTAs after EVAR are crucial to monitor the course of the aneurysm sac and stent-graft behavior, and will be performed many times over the course of a patient’s life. This strategy results in high cumulative CT effective doses. Despite the higher patient age in this cohort, every approach should be undertaken to reduce cumulative radiation risks [28]. To reduce the radiation dose, different imaging strategies should be considered. A recent systematic review proved that MRI is more sensitive than MSCTA for the detection of T2ELs, and may be considered as a complementary imaging modality in negative or uncertain findings at MSCTA [29]. Color-coded duplex-sonography, especially with the use of contrast material [30], has been proposed as an alternative imaging modality [27]; however, imaging results rely on user experience, patient habitus, and the limitations of penetration depth and resolution.

### Limitations

Admittedly, our study has some limitations. First, it is limited by its retrospective nature.

Second, the use of two different MSCTA scanners during the observation period could have influenced T2EL detection rate. Third, a potential limitation could be the lack of native phase images, which might reduce the accuracy of T2EL detection.

## Conclusion

In arterial phase images, non-specialized readers correctly identified T2ELs with a relevant aneurysm sac growth. In addition, the detection of feeding vessels was significantly higher in arterial compared to venous phase images. Therefore, to reduce the radiation dose and potential radiation risks, the elimination of venous phase images in routine follow-up examinations is reasonable.

## Supporting Information

S1 Data Statistical Data. Individual data points.
(SAV)
