# Correction: Type 2 Endoleaks: The Diagnostic Performance of Non-Specialized Readers on Arterial and Venous Phase Multi-Slice CT Angiography

**DOI:** 10.1371/journal.pone.0162431

**Published:** 2016-09-01

**Authors:** Richard Nolz, Ulrika Asenbaum, Julia Furtner, Ramona Woitek, Sylvia Unterhumer, Andreas Wibmer, Alexander Prusa, Christian Loewe, Maria Schoder

The second author’s name is incorrectly reversed. The correct name is Ulrika Asenbaum. The correct citation is: Nolz R, Asenbaum U, Furtner J, Woitek R, Unterhumer S, Wibmer A, et al. (2016) Type 2 Endoleaks: The Diagnostic Performance of Non-Specialized Readers on Arterial and Venous Phase Multi-Slice CT Angiography. PLoS ONE 11(3): e0149725. doi:10.1371/journal.pone.0149725
